# Applying GRADE-CERQual to qualitative evidence synthesis findings–paper 7: understanding the potential impacts of dissemination bias

**DOI:** 10.1186/s13012-017-0694-5

**Published:** 2018-01-25

**Authors:** Andrew Booth, Simon Lewin, Claire Glenton, Heather Munthe-Kaas, Ingrid Toews, Jane Noyes, Arash Rashidian, Rigmor C. Berg, Brenda Nyakang’o, Joerg J. Meerpohl, GRADE-CERQual Coordinating Team, Meghan A. Bohren, Benedicte Carlsen, Ruth Garside, Özge Tuncalp, Megan Wainwright

**Affiliations:** 1 School of Health and Related Research (ScHARR), University of Sheffield, Sheffield, UK; 2 Norwegian Institute of Public Health, Oslo, Norway; 3 Health Systems Research Unit, South African Medical Research Council, Cape Town, South Africa; 4 Unit for Social Welfare Research, Norwegian Institute of Public Health, Oslo, Norway; 5 Cochrane Germany, Medical Center - University of Freiburg, Faculty of Medicine, University of Freiburg, Freiburg, Germany; 6 School of Social Sciences, Bangor University, Bangor, UK; 7 Department of Health Management and Economics, School of Public Health, Tehran University of Medical Sciences, Tehran, Iran; 8 Department of Information, Evidence and Research Department, Eastern Mediterranean Regional Office, World Health Organization, Cairo, Egypt; 9 Department of Community Medicine, University of Tromsø, Tromsø, Norway; 10 Institute of Health & Society, Newcastle University, Newcastle upon Tyne, UK; 11 Centre de Recherche Épidémiologie et Statistique Sorbonne Paris Cité – U1153, Inserm/Université Paris Descartes, Cochrane France, Hôpital Hôtel-Dieu, 1 place du Parvis Notre Dame, 75181 Paris, Cedex 04 France

**Keywords:** Qualitative research, Qualitative evidence synthesis, Systematic review methodology, Research design, Methodology, Confidence, Evidence-based practice, Dissemination bias, Publication bias, GRADE

## Abstract

**Background:**

The GRADE-CERQual (Confidence in Evidence from Reviews of Qualitative research) approach has been developed by the GRADE (Grading of Recommendations Assessment, Development and Evaluation) Working Group. The approach has been developed to support the use of findings from qualitative evidence syntheses in decision-making, including guideline development and policy formulation.

CERQual includes four components for assessing how much confidence to place in findings from reviews of qualitative research (also referred to as qualitative evidence syntheses): (1) methodological limitations, (2) coherence, (3) adequacy of data and (4) relevance. This paper is part of a series providing guidance on how to apply CERQual and focuses on a probable fifth component, dissemination bias. Given its exploratory nature, we are not yet able to provide guidance on applying this potential component of the CERQual approach. Instead, we focus on how dissemination bias might be conceptualised in the context of qualitative research and the potential impact dissemination bias might have on an overall assessment of confidence in a review finding. We also set out a proposed research agenda in this area.

**Methods:**

We developed this paper by gathering feedback from relevant research communities, searching MEDLINE and Web of Science to identify and characterise the existing literature discussing or assessing dissemination bias in qualitative research and its wider implications, developing consensus through project group meetings, and conducting an online survey of the extent, awareness and perceptions of dissemination bias in qualitative research.

**Results:**

We have defined dissemination bias in qualitative research as a systematic distortion of the phenomenon of interest due to selective dissemination of studies or individual study findings. Dissemination bias is important for qualitative evidence syntheses as the selective dissemination of qualitative studies and/or study findings may distort our understanding of the phenomena that these syntheses aim to explore and thereby undermine our confidence in these findings.

Dissemination bias has been extensively examined in the context of randomised controlled trials and systematic reviews of such studies. The effects of potential dissemination bias are formally considered, as publication bias, within the GRADE approach. However, the issue has received almost no attention in the context of qualitative research. Because of very limited understanding of dissemination bias and its potential impact on review findings in the context of qualitative evidence syntheses, this component is currently not included in the GRADE-CERQual approach.

**Conclusions:**

Further research is needed to establish the extent and impacts of dissemination bias in qualitative research and the extent to which dissemination bias needs to be taken into account when we assess how much confidence we have in findings from qualitative evidence syntheses.

**Electronic supplementary material:**

The online version of this article (10.1186/s13012-017-0694-5) contains supplementary material, which is available to authorized users.

## Background

**Fig. 1:**
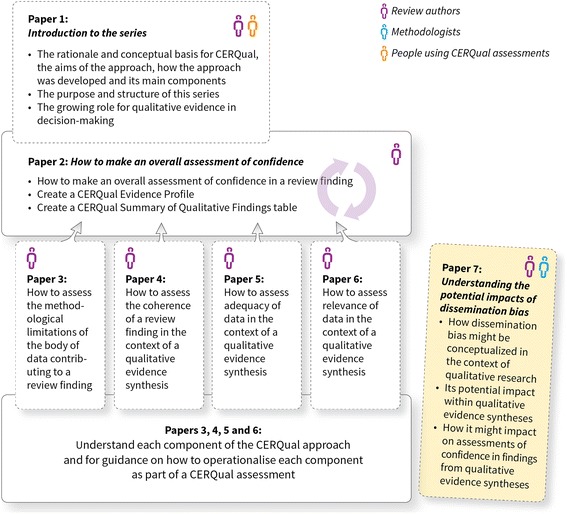
Overview of the GRADE-CERQual series of papers

The GRADE-CERQual (Confidence in Evidence from Reviews of Qualitative research) approach has been developed by the GRADE (Grading of Recommendations Assessment, Development and Evaluation) Working Group. GRADE-CERQual (hereafter referred to as CERQual) currently includes four components for assessing how much confidence to place in findings from reviews of qualitative research (also referred to as qualitative evidence syntheses): (1) methodological limitations, (2) coherence, (3) adequacy of data and (4) relevance. This paper is part of a series (see Fig. 1) and discusses a probable fifth component, dissemination bias and its potential impact on an overall assessment of confidence in a review finding.

## Aims

The aims of this paper are to discuss a definition of dissemination bias in qualitative research and consider how and to what extent it might occur, to explain why dissemination bias may be important in relation to the process and findings of qualitative evidence syntheses, to discuss how dissemination bias might impact on assessments of confidence in findings from qualitative evidence syntheses and to outline an agenda for future research. A complementary paper takes a broader look at dissemination bias in qualitative research and potential lessons from available evidence in the quantitative research arena to inform an understanding of the causes and consequences of dissemination bias in qualitative research [1]. Key definitions for the series are provided in Additional file 1.

## How CERQual was developed

This paper has been developed in collaboration with the GRADE Working Group (www.gradeworkinggroup.org). The initial stages of the process for developing CERQual, which started in 2010, are outlined elsewhere [2]. Since then, we have further refined the current definitions of each component and the principles for application of the overall approach using a number of methods. We used a pragmatic approach to develop our ideas on dissemination bias by consulting the literature on this topic, including searching MEDLINE and Web of Science to identify and characterise the existing literature discussing or assessing dissemination bias in qualitative research and its wider implications (Nyakang’o SB, Booth A, Meerpohl JJ, Glenton C, Lewin S, Berg RC, Munthe-Kaas HM, Toews I, for the GRADE-CERQual DissQuS Subgroup: Describing non-dissemination and dissemination bias in qualitative research: a mapping review, In preparation); talking to experts in dissemination bias and qualitative evidence synthesis in a number of workshops; and developing consensus through multiple face-to-face CERQual Project Group meetings and teleconferences. We also undertook an online survey of researchers, journal editors and peer reviewers within the qualitative research domain on the extent, awareness and perceptions of dissemination bias in qualitative research [3]. The methods used to develop CERQual are described in more detail in the first paper in this series [4].

## Dissemination bias and qualitative research

Dissemination bias (which encompasses publication bias) has been studied and discussed extensively in the context of randomised controlled trials and other effectiveness studies. The impacts of dissemination bias on the findings of systematic reviews of the effects of interventions have also received considerable attention [5–7]. Acknowledging this empirical evidence base, the GRADE for effectiveness approach includes dissemination bias, under the label of ‘publication bias’, as one of the five domains considered when assessing the certainty of evidence, noting that, ‘Even if individual studies are perfectly designed and executed, syntheses of studies may provide biased estimates because systematic review authors or guideline developers fail to identify studies’ ([8], p. 1278). Non-identification of studies may occur, for example, because effectiveness studies with negative findings have a lower chance of being disseminated than studies that report positive findings [9].

**Table 1:** Defining dissemination bias in the context of qualitative research

GRADE-CERQual definition of dissemination bias in the context of qualitative research	A systematic distortion of the phenomenon of interest due to selective dissemination of qualitative studies or the findings of qualitative studies
GRADE for effectiveness definition of publication bias	A systematic under-estimation or an over-estimation of the underlying beneficial or harmful effect due to the selective publication of studies [31]
OPEN framework of (non-) dissemination of research findings	The approach includes three parts: 1. Issues that need to be considered when exploring possible biases due to selective dissemination of research findings (What?) 2. Stakeholders who could assume responsibility for the various stages of conducting a clinical trial and disseminating clinical trial documents (Who?) 3. Motivations that may lead the various players to disseminate study findings selectively, thereby introducing bias in the dissemination process (Why?) [17]
Cochrane definition of publication bias	The *publication* or *non-publication* of research findings, depending on the nature and direction of the results [18]
Cochrane definition of outcome reporting bias	The *selective reporting* of some outcomes but not others, depending on the nature and direction of the results [18]

The issue of dissemination bias has received little attention in the context of qualitative research [2]. This leaves a major gap in our understanding of how dissemination bias might impact on the findings of qualitative evidence syntheses and on assessments of confidence in these findings. Because of our limited understanding of the issue, dissemination bias is not currently included in the CERQual approach. This paper therefore differs from the others in this series in that we do not provide guidance on applying this potential component of the CERQual approach. Rather, we focus here on how dissemination bias in the context of qualitative research might be conceptualised and why it might be important to assess its potential impact within qualitative evidence syntheses. As discussed in the first paper in this series [4], we have adopted the ‘subtle realist’ position [10] in our approach to qualitative evidence synthesis and the development of CERQual. Viewed from this perspective, the systematic omission of individual findings or whole studies, and the potential threat that this poses to both the richness and completeness of our understanding of a phenomenon, is a methodological challenge that we need to address rather than an insurmountable obstacle to qualitative evidence synthesis.

Some readers may be surprised by our use of the term ‘bias’ in the context of qualitative research. Indeed, this was the subject of considerable discussion within our group given the term’s association with the positivist paradigm. We would argue that ‘bias’ is sufficiently established within the qualitative, interpretivist paradigm to be a useable term in this context. In their text on qualitative methods, Bloor and Wood define bias as, ‘Any influence that distorts the results of a research study’. They go on to note that, ‘Bias may derive either from a conscious or unconscious tendency on the behalf of the researcher to collect data or interpret them in such a way as to produce erroneous conclusions that favour their own beliefs or commitments’ ([11], p. 21). We use the term bias in a similar way, but rather than applying it to the conduct of qualitative studies, we focus on the dissemination of the findings of qualitative studies.

## What is dissemination bias in the context of qualitative research?

We have defined dissemination bias in the context of qualitative research as ‘a systematic distortion of the phenomenon of interest due to selective dissemination of qualitative studies or the findings of qualitative studies’. There are several important elements in this definition: firstly, the term ‘phenomenon of interest’ refers to the issue that is the focus of qualitative inquiry. The phenomenon of interest may relate to an intervention, a condition/situation or an issue, and is often outlined in the question or scope underlying the primary qualitative study or qualitative evidence synthesis [2].

Secondly, we use the term ‘systematic distortion’ to indicate that we are concerned with a distortion of our understanding of the phenomenon of interest that occurs because certain groups of study findings are systematically less easily accessible or available (rather than study findings not being accessible or available in a random way). These groups of study findings may be less accessible or available in part or in their entirety. For instance, if studies with findings regarding a particularly sensitive aspect of the phenomenon are seldom submitted for publication, that aspect of the phenomenon will be poorly understood. As a consequence, our understanding of the phenomenon as a whole will be incomplete. Of course, the findings of many qualitative studies are never disseminated, or are only disseminated in part [3, 12]. While this is unethical [13] and leads to research waste [14], it will not result in bias if the non-dissemination is random (and thus will not distort our understanding of the phenomenon of interest in a systematic or consistent way).

Another way of looking at this is that the importance of non-dissemination depends on the extent to which the study findings that have been disseminated regarding a phenomenon encompass the full range of findings from those studies. If the range of study findings disseminated is similar to all of the findings identified in the studies, systematic distortion is unlikely. However, if the findings that have been disseminated are consistently different from the full universe of findings that have been identified from primary research, systematic distortion of the phenomenon of interest is likely to occur [9].

Thirdly, we use the term ‘dissemination bias’ rather than ‘publication bias’ to acknowledge the wide range of ways to disseminate the findings of qualitative studies beyond publication in an indexed journal. ‘Publication’ is also increasingly difficult to define given the variety of electronic and alternative formats through which the findings of qualitative studies can be made available, such as institutional websites, registries of studies and book chapters. We are therefore more concerned with the non-availability or non-accessibility of qualitative study findings rather than only whether they have been formally published or not [9]. If study findings are not disseminated in an accessible way, then dissemination bias might result. In addition, our definition of dissemination bias does not extend to differential ‘uptake’ of the findings of qualitative studies which relates to the behaviours of users rather than to those of the evidence producers.

Fourth, we mention both qualitative studies and the findings of qualitative studies in our definition of dissemination bias. This is to indicate that we are concerned both with the selective dissemination of entire studies and of particular findings from studies. While the selective dissemination of entire studies is more widely discussed in the scientific literature, multiple factors may explain why the study findings themselves may be disseminated selectively. For example, particular study findings that are unpalatable to governments, research commissioners or research funders may not be disseminated ([11], p. 22). Alternatively, researchers may be asked to earmark available space within their manuscript to study findings that are considered more newsworthy, by implication ‘truncating’, or even omitting, dissemination of other aspects of the phenomenon of interest ([11], p. 22) [15]. Similarly, qualitative study findings that run counter to a mainstream understanding of a phenomenon, or ways of describing a phenomenon, may be removed from a paper on the request of the peer reviewers or editors and, consequently, may not be disseminated [16].

Our definition of dissemination bias is compatible both with recent broader work to develop a consistent and comprehensive approach to defining the non-dissemination of research findings [17] and with the definitions of publication bias used by the GRADE for effectiveness approach [8] and Cochrane [18] (Table 1).

## When might dissemination bias arise in the process of disseminating the findings of qualitative studies?

Decisions made at numerous points in the process of disseminating the findings of qualitative studies may lead to selective dissemination which may, in turn, result in dissemination bias. Table 2 illustrates some of the decision points, each of which could be unpacked in more detail through examining the contributing decisions. This table simply seeks to describe possible decisions impacting on dissemination without exploring underlying mechanisms or the contexts under which these decisions may be considered more or less appropriate. Decisions taken by the authors of primary studies or qualitative evidence syntheses also impact, for example, on which primary research is prioritised, how this research is conducted, which types of studies are included in qualitative evidence syntheses and which interpretations are favoured in the synthesis process. However, in the context of assessing how much confidence to place in the findings of qualitative evidence syntheses, we are primarily concerned with decisions made in the process of disseminating individual study findings. It is these decisions that may result in dissemination bias and, consequently, in systematic distortion of the phenomenon of interest.

**Table 2:** When dissemination bias may arise in the process of writing up and disseminating the findings of qualitative studies

Stage of the dissemination process	How dissemination bias may arise
Funder/commercial/policy interests	Studies or study findings not disseminated because of funder interests, commercial interests or other interests related to a policy process
Decision to write/submit for publication	• Study findings contrary to popular opinion or practice more/less likely to be written up or disseminated • Most novel or striking study findings selected for publication • Findings of unfunded studies less likely to be submitted for publication
Decisions on which themes/findings to include or emphasise in study reports	Study authors favour particular interpretations
Choice of dissemination strategy	• Study authors choose avenue/s to disseminate the study findings (e.g., to which journal to submit the paper) that result in findings being less available • Studies in some languages more likely to be published in non-indexed journals and therefore their findings are less available
Editorial policies of journals and other dissemination forums	• Journal editors/peer reviewers favour studies reporting findings focusing on particular issues • Word limits make full publication of findings less likely
Inclusion in databases	• Particular study findings more/less like to be found if the studies reporting these, or the journals typically publishing these, are more/less likely to be included in databases and therefore to be retrieved

This table does not intend to provide a comprehensive overview of all the routes through which dissemination bias may arise in writing up and disseminating the findings of qualitative studies

## Why might dissemination bias be important in a CERQual assessment?

In the CERQual approach, all review findings start off as ‘high confidence’ and this assessment is then modified if there are concerns regarding any of the CERQual components. This starting point of ‘high confidence’ reflects a view that each review finding should be seen as a reasonable representation of the phenomenon of interest unless concerns are identified to weaken this assumption. Dissemination bias is one concern that may weaken this assumption as the synthesis findings regarding the phenomenon of interest may be distorted if the findings from the group of available and included studies are systematically unrepresentative of the full body of research that has been conducted. As with the existing four CERQual components, the intention is not to exclude potentially valuable insights from studies on the basis of an individual CERQual component judgement or the overall CERQual assessment. Rather, the intention is simply to take into account considerations that impact on confidence in the review findings.

## What is the extent of dissemination bias in qualitative research?

Empirical evidence on the extent of dissemination bias in qualitative research, and how it varies across the different fields in which qualitative research is undertaken, is very limited. To our knowledge, only one study has explored empirically the extent of non-dissemination of qualitative research [12]. This study of a cohort of 224 abstracts examined publications emerging from qualitative studies presented at a medical sociology conference. It reported an overall publication rate of only 44.2%--a figure similar to that for quantitative biomedical research presented at conferences [19, 20]. The authors observed that non-publication appeared to be related to the quality of reporting, including whether the research question was outlined and the methods and findings described. This suggests a mechanism by which qualitative studies that do not show ‘clear, or striking, or easily described findings simply disappear from view’ ([12] p. 552), with the implication that qualitative evidence syntheses that only rely on published papers may be subject to ‘qualitative publication bias’ ([12] p. 552).

The GRADE-CERQual Project Group has conducted two research projects to widen our understanding of the nature and extent of non-dissemination and dissemination bias in this field. The first study, a mapping review, aims to identify and document the existing literature discussing dissemination bias and related effects in qualitative research. The second study, a cross-sectional survey, aimed to explore stakeholders’ views and experiences of, and reasons for, the non-dissemination of qualitative research studies and individual study findings [3]. The survey findings suggest that the proportion of unpublished qualitative studies and individual findings is substantial and comparable to the extent of non-dissemination of studies using quantitative methods. Considerable further research is needed on both the extent of dissemination bias in qualitative research, including partial reporting of research findings, and the factors that affect this––we discuss this research agenda in more detail below and in a complementary paper [1].

## When might one suspect that dissemination bias may be present?

At present, there is no methodological guidance available on how to assess the possibility and impacts of dissemination bias in the context of a qualitative evidence synthesis. Observations that may lead a review team to suspect dissemination bias include:

- Evidence that primary research has been carried out in relation to the synthesis question (for example, evidence that studies have been funded or presented at conferences, the availability of a protocol, or details reported in the methods section of a study) but the full set of study findings are not available (for instance, as a journal article or report)
- Findings from available studies reflect only a limited range of participants, settings, time periods, aspects of the phenomena of interest or conceptual or theoretical perspectives when it is likely that a wider range of contexts, time periods, phenomena or perspectives have been considered in research in the area
- Findings are available in languages that are not accessible to the review team
- Available studies all indicate strong formative input from funders of qualitative research, editors of journals publishing qualitative research or other stakeholders with particular interests in particular types of study findings
- Differences in completeness or emphasis that are revealed when comparing findings published in a journal with a corresponding fuller account, such as a thesis or book chapter

Methods need to be developed for exploring whether the findings of a synthesis have been distorted systematically by dissemination bias, and this is a key focus for further research by the GRADE-CERQual Project Group. However, there are numerous reasons why it may be difficult to identify the effects of dissemination bias within qualitative evidence syntheses. Firstly, the contribution of an individual qualitative study to a particular interpretation cannot easily be discerned [21]. Secondly, the occurrence of a finding from a single study in isolation is not in itself an indicator of the presence of bias as it may simply reflect a divergent or disconfirming case [22]. Thirdly, unlike in quantitative research, procedures for estimating or projecting the total population of relevant studies have not yet been developed [23]. Finally, reflexivity--that is, looking critically at the impacts of the review authors on all aspects of a synthesis--is usually encouraged within a frame of what has been included in a synthesis and not in terms of what may have been omitted [24].

A growing number of qualitative evidence syntheses are reporting consideration of the impacts of dissemination bias on the studies identified for the synthesis and, to some extent, on the review findings as a whole [25–30]. This is an important first step in relation to documenting possible dissemination bias and identifying examples of its potential impacts. In Table 3, we outline a research agenda for exploring dissemination bias in qualitative research.

**Table 3:** A research agenda for exploring the impacts of dissemination bias on the findings of qualitative evidence syntheses

• Explore the impacts of dissemination bias on the findings of qualitative evidence syntheses	
• Develop methods for identifying dissemination bias that can used by those conducting qualitative evidence syntheses	
• Explore the interactions, if any, of dissemination bias and the other components of the CERQual approach (i.e. methodological limitations, coherence, adequacy and relevance)	
• Identify potential interventions to reduce the impact of dissemination bias within qualitative evidence syntheses and evaluate their likely effectiveness	

## Conclusions

Important goals of the GRADE-CERQual Project Group are to improve understanding of how dissemination bias might occur in qualitative research; its likely impact on the degree of confidence that can be placed in the findings of qualitative evidence syntheses; and whether and how to include dissemination bias as a fifth component of the CERQual approach. We also hope that an improved understanding of dissemination bias may, in the longer term, lead study authors, journal editors, peer reviewers and other stakeholders to devise strategies to minimise the impact of such biases.

## Open peer review

Peer review reports for this article are available in Additional file 2.

## Additional files

Additional file 1: Key definitions relevant to CERQual. (PDF 619 kb)

Additional file 2: Open peer review reports. (PDF 99 kb)
