# Supplementary material for: Applying GRADE-CERQual to qualitative evidence synthesis findings–paper 7: understanding the potential impacts of dissemination bias
Source: Implement Sci. 2018 Jan 25;13(Suppl 1):12. doi: 10.1186/s13012-017-0694-5 (PMC5791043; doi:10.1186/s13012-017-0694-5)
Supplement: Supplementary file 2 — Open peer review reports. (PDF 99 kb) [file 13012_2017_694_MOESM2_ESM.pdf]

Open Peer Review reports

Original Submission		
21 Oct 2016	Submitted	Original Manuscript
19 Jan 2017	Reviewed	Reviewer Report - GJ Melendez-Torres

In the main, I thought this paper was an explicit, clear and usable treatment of how to examine methodological limitations in a review finding. It was a privilege to review this paper.

There is one area that could do with some clarification. The necessarily intersubjective aspects of qualitative metasynthesis have implications for how we regard methodological limitations within and between a body of evidence, and how strongly we are able to assert a position on what methodological limitations are. At the moment, it primarily appears as if the paper primarily locates methodological limitations in data collection and lack of reflexivity. But there is a diversity of methodological limitations that may occur in qualitative research... for example, when one method is stated but the findings 'read' like a different method. (In my experience, this happens a lot with an ostensibly grounded theory method yielding something that actually reads like a thematic analysis.) Similarly, the criteria used to evaluate a phenomenology will not look like the criteria used to evaluate a framework analysis. So, how does assessment of methodological limitations account for diversity of method (and thus, diversity of quality markers), and how does an analysis of methodological limitations account for more 'advanced' issues in methods than just horses for courses?

Finally, one minor thought is that it may be useful to tie in the discussion of how findings may rely on low-quality studies with the practice of 'sensitivity analysing' findings in reviews to account for variable quality in studies contributing.

17 Mar 2017	Reviewed	Reviewer Report - Helen Elsey

This is a useful paper in explaining the process needed for assessing the methodological strengths/limitations of the findings of qualitative systematic reviews.

It would be helpful if figure 1 could include some bullet points of the key factors under each of the components. This could highlight when there are areas of overlap, as mentioned in the text.
Table 1 is helpful in providing examples of the kind of methodological limitations and how they might affect our confidence in review findings.

The steps are clear and logical. What is difficult to grasp is how much these should be done by the reviewer of a review, and how much should be expected to have already been done by the reviewers of the original studies. I.e. is your recommendation to those doing qualitative reviews to present transparently their assessment of the methodological strength of each of their review findings, or are you suggesting that the reviewer of the review should go back to the original studies and do this themselves?

Perhaps, the point is that most reviews don't present this assessment of quality under each finding, therefore the reviewer of the review has to do it themselves.

Understanding whether you 3 steps are aimed at reviewers, or reviewers of reviews, would be clearer if there was more detail about the process that you went through to come up with these 3 steps. These are mentioned in the abstract, but no further detail is given i.e. when you did your literature search were you looking for qualitative synthesis to see whether they had presented their own assessment of the methodological limitations of the body of data? is this commonly done, and if so how? is this what your were trying to find out from the 'research communities' and the project group meetings? Greater clarity on who was contacted, who was part of the project group, what you asked each group to do and how they inputted to the development of the steps. When you tested CERQual on several (could you give the actual number?) of qualitative evidence synthesis how did it go? Does it work better with some types of subject areas, or types of review than others?
Providing greater detail on the process you went through and who your are aiming your recommended 'steps' at (reviewers or reviewers of reviews) would be helpful.

24 Jun 2017 Author responded

Author comments - Heather Munthe-Kaas

**Peer reviewer comments**	**Responses**
**Reviewer #1:**	
In the main, I thought this paper was an explicit, clear and usable treatment of how to examine methodological limitations in a review finding. It was a privilege to review this paper.	Thank you
There is one area that could do with some clarification. The necessarily intersubjective aspects of qualitative metasynthesis have implications for how we regard methodological limitations within and between a body of evidence, and how strongly we are able to assert a position on what methodological limitations are. At the moment, it primarily appears as if the paper primarily locates methodological limitations in data collection and lack of reflexivity. But there is a diversity of methodological limitations that may occur in qualitative research... for example, when one method is stated but the findings 'read' like a different method. (In my experience, this happens a lot with an ostensibly grounded theory method yielding something that actually reads like a thematic analysis.) Similarly, the criteria used to evaluate a phenomenology will not look like the criteria used to evaluate a framework analysis. So, how does assessment of methodological limitations account for diversity of method (and thus, diversity of quality markers), and how does an analysis of	We have updated this section according to newly published advice from Cochrane Qualitative and Implementation Methods Group. This should cover the reviewer's comments. We have also included a new table (Table 2) with examples to illustrate different types of methodological limitations that may influence our confidence in our review finding.

methodological limitations account for more 'advanced' issues in methods than just horses for courses?	
Finally, one minor thought is that it may be useful to tie in the discussion of how findings may rely on low-quality studies with the practice of 'sensitivity analysing' findings in reviews to account for variable quality in studies contributing.	In paper 2 we have discussed this issue more thoroughly. If you have a finding from a low and high quality study, you wouldn't rate down – see paper 2 for a more comprehensive discussion.
**Reviewer #2:**	
This is a useful paper in explaining the process needed for assessing the methodological strengths/limitations of the findings of qualitative systematic reviews.	Thank you
It would be helpful if figure 1 could include some bullet points of the key factors under each of the components. This could highlight when there are areas of overlap, as mentioned in the text.	Thank you for the suggestion. However, we don't want to crowd the diagram. Paper 2 has a more comprehensive discussion about areas of overlap.
Table 1 is helpful in providing examples of the kind of methodological limitations and how they might affect our confidence in review findings.	Thank you
The steps are clear and logical. What is difficult to grasp is how much these should be done by the reviewer of a review, and how much should be expected to have already been done by the reviewers of the original studies. I.e. is your recommendation to those doing qualitative reviews to present transparently their assessment of the methodological strength of each of their review findings, or are you suggesting that the reviewer of the review should go back to the original studies and do this themselves? Perhaps, the point is that most reviews don't present this assessment of quality under each finding, therefore the reviewer of the review has to do it themselves.	- These steps are not intended for the reviewer of a review.<br>- We have added a small paragraph emphasizing that the guidance in this article is intended for systematic review authors applying CERQual to findings from a review they conducted (unless otherwise specified).
Understanding whether you 3 steps are aimed at reviewers, or reviewers of reviews, would be clearer if there was more detail about the process that you went through to come up with these 3 steps. These are mentioned in the abstract, but no further detail is given i.e. when you did your literature search were you looking for qualitative synthesis to see whether they had presented their own assessment of the methodological	Thank you for this feedback. In response:<br>- These steps are not intended for the reviewer of a review.<br>- CERQual is not a critical appraisal tool for qualitative evidence syntheses<br>- We have expanded on the methods for developing this component in the methods section of the paper.

limitations of the body of data? is this commonly done, and if so how? is this what your were trying to find out from the 'research communities' and the project group meetings? Greater clarity on who was contacted, who was part of the project group, what you asked each group to do and how they inputted to the development of the steps. When you tested CERQual on several (could you give the actual number?) of qualitative evidence synthesis how did it go? Does it work better with some types of subject areas, or types of review than others?	
Providing greater detail on the process you went through and who your are aiming your recommended 'steps' at (reviewers or reviewers of reviews) would be helpful.	See above

Resubmission

24 Jun 2017 Submitted Manuscript version 2

9 Oct 2017 Author responded Author comments - Heather Munthe-Kaas

General comments from the series editor	Author responses and changes made
Thanks for providing more methodological detail in the overview and subsequent papers. There are still some areas where it would be better if you could provide further details to reflect the amount of international developmental work undertaken e.g. databases searched, timeframes, how literature reviewed etc.	We have added further detail to the overall methods description in paper 1 of the series. Specifically, we have:<br>- Included the years during which we ran workshops and seminars to obtain feedback on CERQual, and the numbers of workshops and presentations undertaken<br>- Specified the period during which small group feedback sessions were run<br>- Specified the number of CERQual users and Project Group members interviewed<br><br>In the component papers (papers 3-6), we have noted that the literature searches that we undertook were informal in nature, as follows (example from paper 5):<br>"When developing CERQual's adequacy component, we undertook informal searches of the literature, including Google and Google Scholar, for definitions and discussion papers related to the

		concept of adequacy and to related concepts such as data quantity, sample size and data saturation."<br><br>We have also elaborated on the methods used to develop the content of paper 7 – please see below.
	Ethics statements. Papers state that no humans were involved. Suggest amending to reflect consensus approach, interviews and questionnaires undertaken.	As we did not undertake formal data collection with people – all data collection was informal, in the context of training workshops, presentations and assessments of use of the approach, we have changed the ethics approval and consent to participate statements to the following:<br>"Not applicable. This study did not undertake any formal data collection involving humans or animals."
	Titles and papers could reflect paper nth of # part in a series.	We have changed all titles to the following format, as agreed earlier (example from paper 1):<br>'Applying GRADE-CERQual to qualitative evidence synthesis findings – paper 1 of 7: Introduction to the series'
	State of the art has been removed from paper 6 but not all of the other papers in the series.	'State of the art' has been removed from all papers in the series.
	The new figure outlining the process is a good addition. As a reader I would have found it easier to read papers 3-6/7 before reading paper 2.	As discussed by email with Liz Glidewell, we had a very long debate within the group about this and concluded that there is no perfect order because paper 2 (overall assessment) and papers 3-6 (components) need to be seen together. We placed 'overall assessment' before the component papers as we felt that readers needed to understand what they were working towards before understanding each component. We feel that it would be best to keep the order as it is, but have made the following changes to assist readers:<br><br>Papers 2, 3, 4, 5 and 6:<br>We have inserted text along the lines of the following (example from paper 2 (p6): 'These component papers are closely related to this paper on making an overall CERQual assessment of confidence and creating a Summary of Qualitative Findings table. We have placed this paper before the four CERQual component papers as we think that it will be helpful for readers to understand how the component assessments will be used before discussing the details of how to apply each component.'<br><br>Papers 2, 3, 4, 5 and 6:

	We have included in each paper an additional table that brings together all of the key definitions from each of the papers.
Do you still want to publish paper 7 as a standalone or incorporate it into the overview along with the other ongoing research?	Yes, we feel that it works best as a standalone paper.
Would the figure in the introduction outlining the process work better across all papers in the series as it contains more information than the figure just outlining the 4 and probable 5th component?	Thanks for this very helpful suggestion which we have implemented across all of the papers.
**1. <u>Introduction</u>**	
The lack of such methods constrains the use of…suggest reframing to "methods may constrain".	Change made
"The CERQual approach is intended to be applied to well conducted syntheses." Could this be confusing to those applying the four components? Isn't CERQual designed to provide evidence of confidence in a well conducted syntheses?	We have not found this to be confusing in our interactions with users of CERQual. We feel that there would be little point in applying CERQual to a synthesis that has been poorly conducted as the findings of such a synthesis are unlikely to be reliable and the synthesis is unlikely report transparently the methods used or to include sufficient information on the primary studies to allow a CERQual assessment to be undertaken. We take the same approach in relation to GRADE for effectiveness, for the same reasons. The problem is sometimes colloquially called 'garbage in-garbage out'!
The section "Applying CERQual across types of qualitative data and syntheses methods". Would this be better placed after outlining how CERQual was developed?	We agree and have moved this section.
"supported other teams". Can you say any more about the scale or settings involved?	We have provided more detail as follows:<br>"Thirdly, we applied the CERQual approach within diverse qualitative evidence syntheses in the areas of health and social care [6-8, 26-33] and also supported other teams in using CERQual by providing guidance through face-to-face or virtual training meetings and commenting on draft Summaries of Qualitative Findings tables. At least ten syntheses were supported in this way (for example, [34, 35])."
Can you provided further detail about the questionnaire and qualitative interviews?	We have now provided further detail in the text and added an additional file listing the questions covered. The revised text reads as follows:

	"We then gathered structured feedback from early users of CERQual through an online feedback form that was made available to all CERQual users and through short individual discussions with six members of review teams and two members of the CERQual Project Group. The questions included in the online feedback form and individual discussions are available in Additional File X."
Summarise important areas for methodological research from table 4 in text for the readers ease?	We have revised the text as follows:<br>"Table 4 identifies several important areas for further methodological research, including how to apply CERQual in syntheses that include qualitative and quantitative data; how best to present CERQual assessments together with other kinds of evidence; ways of applying CERQual to syntheses of sources that have not used formal qualitative research procedures; and whether CERQual requires adaptation for application to more interpretive synthesis outputs, such as logic models."
**2. Making an overall assessment and summary of qualitative findings**	
Should the paragraph describing the four levels and rating down on p12 be moved to p10 under the 4 bulleted levels of concern?	This change has been made.
Place the text relating to variation in assessors after the text outlining who should undertake an assessment?	This change has been made.
Table 5. typo in component t missing.<br><br>Should you advise assessors to report how they've handled variation in levels of concern?	This typo has been corrected.
**3. Methodological limitations – problems design or conduct of primary studies**	
Consider adding a brief description of the Evidence Profile to p12.	Ok. We have now added the following parentheses describing the evidence profile on page 12 following the sentence: "Where you have concerns about methodological limitations, describe these concerns in the CERQual Evidence Profile in sufficient detail to allow users of the review findings to understand the reasons for the assessments made (The Evidence Profile presents each review

	finding along with an explanation of its CERQual assessment)”
Link in text to table 2?	We have now added the following on page 9: “See Table 2 for an outline of areas where further work is needed with respect to critical appraisal tools for qualitative research.”
**4. Coherence – How well finding supported by body of evidence 3500 3429**	
Consider adding a brief description of the Evidence Profile to p13.	We have added a brief description of the evidence profile on page 12:<br>“Where you have concerns about coherence, you should describe these concerns in the CERQual Evidence Profile in sufficient detail to allow users of the review findings to understand the reasons for the assessments made. The Evidence Profile presents each review finding along with the assessments for each CERQual component, the overall CERQual assessment for that finding and an explanation of this overall assessment. For more information, see the second paper in this series [19].”
**5. Adequacy of data – degree of richness and quantity of data 3500 2507**	
Consider contacting authors for further information as in other assessments?	We have added the following information to lines 204-205:<br><br>“An overview of the number of studies from which this data originated, and where possible, the number of participants or observations. Information about the number of participants or observations supporting each finding may be difficult to gain from the individual studies. While most studies describe the number of participants they included in their study overall or give some indication of the extent of their observations, they may be less clear about how well represented participants are in different themes and categories. You can contact

	study authors for additional information, but they may not be able to readily provide this level of detail. In these cases, this lack of information should be noted, and your assessment of data adequacy will have to be made based on the information available."
The sentence "For a description on descriptive and explanatory findings…" isn't embedded.	We have moved this sentence to lines 232-233.
Consider adding a brief description of the Evidence Profile to p12.	We have added the following information to lines 277-279:<br><br>The Evidence Profile presents each review finding along with the assessments for each CERQual component, the overall CERQual assessment for that finding and an explanation of this overall assessment.
**6. Relevance – extent applicable to context (perspective or population, phenomenon of interest, setting) of review question 3500 3551**	
I found a lot of the text more relevant to conducting a review than the CERQual assessment e.g. using theories and frameworks, how and when the review question should be developed, the pre-specification of sub-groups, strategies for identifying and selecting studies, trade-offs in searching.	Relevance is the only CERQual component that links directly to the review question. All the issues raised by the Editor need to be taken into consideration at the review design stage. We make this clear in the manuscript. See P6:<br><br>*'Relevance is the CERQual component that is anchored to the context specified in the review question. How the review question and objectives are expressed, how a priori subgroup analyses are specified, and how theoretical considerations inform the review design are therefore critical to making an assessment of relevance when applying CERQual.'*<br><br>See page 11: *'When assessing relevance, you should reflect on how the sample was located and on the underpinning principles that determined its selection….'*

Word missing p13 "You should if possible, that this"	Sincere apologies, this typo was corrected previously but the corrected draft was not uploaded last time.
Is it possible to comment on how the levels of concern map onto the different threats to relevance 'partial', 'indirect' and 'unclear'?	Tables 3, 4, 5 and 6 provide visual examples. Sincere apologies, these tables may not have been uploaded in error last time.
**7. Dissemination bias – selective dissemination of studies or findings 2000 2455**	
Methodological details e.g. 'consulting relevant literature' and 'additional empirical work'	We have added further detail as follows:<br><br>Abstract:<br>"We developed this paper by gathering feedback from relevant research communities, searching MEDLINE and Web of Science to identify and characterize the existing literature discussing or assessing dissemination bias in qualitative research and its wider implications, developing consensus through project group meetings, and conducting an online survey of on the extent, awareness and perceptions of dissemination bias in qualitative research."<br><br>Main text:<br>"We used a pragmatic approach to develop our ideas on dissemination bias by consulting the literature on this topic, including searching MEDLINE and Web of Science to identify and characterize the existing literature discussing or assessing dissemination bias in qualitative research and its wider implications [3]; talking to experts in dissemination bias and qualitative evidence synthesis in a number of workshops; and developing consensus through multiple face-to-face CERQual Project Group meetings and teleconferences. We also undertook an online survey of researchers, journal editors and peer reviewers within the qualitative research domain on

			the extent, awareness and perceptions of dissemination bias in qualitative research [4]."
**Resubmission 2**			
9 Oct 2017	Submitted	Manuscript version 3	
**Publishing**			
17 Oct 2017	Editorially accepted		

How does Open Peer Review work?

Open peer review is a system where authors know who the reviewers are, and the reviewers know who the authors are. If the manuscript is accepted, the named reviewer reports are published alongside the article. Pre-publication versions of the article and author comments to reviewers are available by contacting info@biomedcentral.com. All previous versions of the manuscript and all author responses to the reviewers are also available.

You can find further information about the peer review system here.
